# Supplementary material for: Vitamin A Status in Preterm Infants Is Associated with Inflammation and Dexamethasone Exposure
Source: Nutrients. 2023 Jan 14;15(2):441. doi: 10.3390/nu15020441 (PMC9861363; doi:10.3390/nu15020441)
Supplement: Supplementary file 1 [file nutrients-15-00441-s001.zip › Supplementary table S1.pdf]

Table S1. Details on vitamin A biochemistry and intake according to dexamethasone group

	**Inclusion**		**Day 7**		**Day 28**[a]		**Week 36 PMA**	
	No-DEXA (n=58)	DEXA (n=41)	No-DEXA (n=53)	DEXA (n=43)	No-DEXA (n=41)	DEXA (n=39)	No-DEXA (n=48)	DEXA (n=38)
RBP4, µg/mL	14.3 (11.5, 19.2)	14.8 (13.1, 19.5)	18.1 (14.4, 23.2)	14.9 (12.2, 19.5)	16.7 (12.6, 20.4)**	30.1 (20.7, 38.1)**	13.8 (9.9 (16.3)	13.7 (11.7, 17.8)
Retinol, µmol/L	0.53 (0.39, 0.74)	0.55 (0.39, 0.69)	0.66 (0.51, 0.86)**	0.42 (0.30, 0.59)**	0.56 (0.41, 0.74)**	1.0 (0.74, 1.5)**	0.47 (0.34, 0.57)	0.47 (0.35, 0.69)
< 0.7, n (%)	42 (72)	31 (76)	32 (60)*	38 (88)*	29 (71)**	8 (21)**	41 (85)	31 (82)
< 0.35, n(%)	9 (16)	7 (17)	3 (6)*	13 (30)*	2 (5)	1 (3)	14 (29)	9 (24)
Vitamin A intake[b], µg/kg/day								
Total			256 (201, 298)**	208 (175, 247)**	955 (840, 1046)	966 (814, 1034)	755 (637, 848)	771 (680, 872)
Enteral			109 (48, 169)**	47 (32, 98)**	940 (822, 1043)	961 (757, 1023)		
Parenteral			143 (122, 160)	146 (132, 160)	11 (4, 33)	13 (5, 36)		
< RDA[c], n%			49 (92)	42 (98)	2 (5)	1 (3)	3 (6)	1 (3)

DEXA, dexamethasone; PMA, postmenstrual age; RBP4, retinol binding protein-4; RDA, recommended daily allowance.

Values are presented as n (%) and median (IQR).

[a]3 cases were excluded due to days off protocol > 4 days (1 case in the DEXA group)

[b]Vitamin A intake between visits. Only infants with available data on biochemical vitamin A status were included in nutrient analysis.

[c]RDA based on ESPGHAN enteral guidelines 2022 (400-1000 µg vitamin A/kg/day)

*p<0.01, **p<0.001
