# Supplementary material for: Vitamin A Status in Preterm Infants Is Associated with Inflammation and Dexamethasone Exposure
Source: Nutrients. 2023 Jan 14;15(2):441. doi: 10.3390/nu15020441 (PMC9861363; doi:10.3390/nu15020441)
Supplement: Supplementary file 1 [file nutrients-15-00441-s001.zip › Supplementary.pdf]

Supplementary

Table of contents

Figure S1. Longitudinal measures of retinol concentrations according to randomization group

Figure S2. Associations between first week CRP levels and retinol concentrations

Figure S3. Longitudinal measures of retinol concentrations according to BPD group

Table S1. Details on vitamin A biochemistry and intake according to dexamethasone group

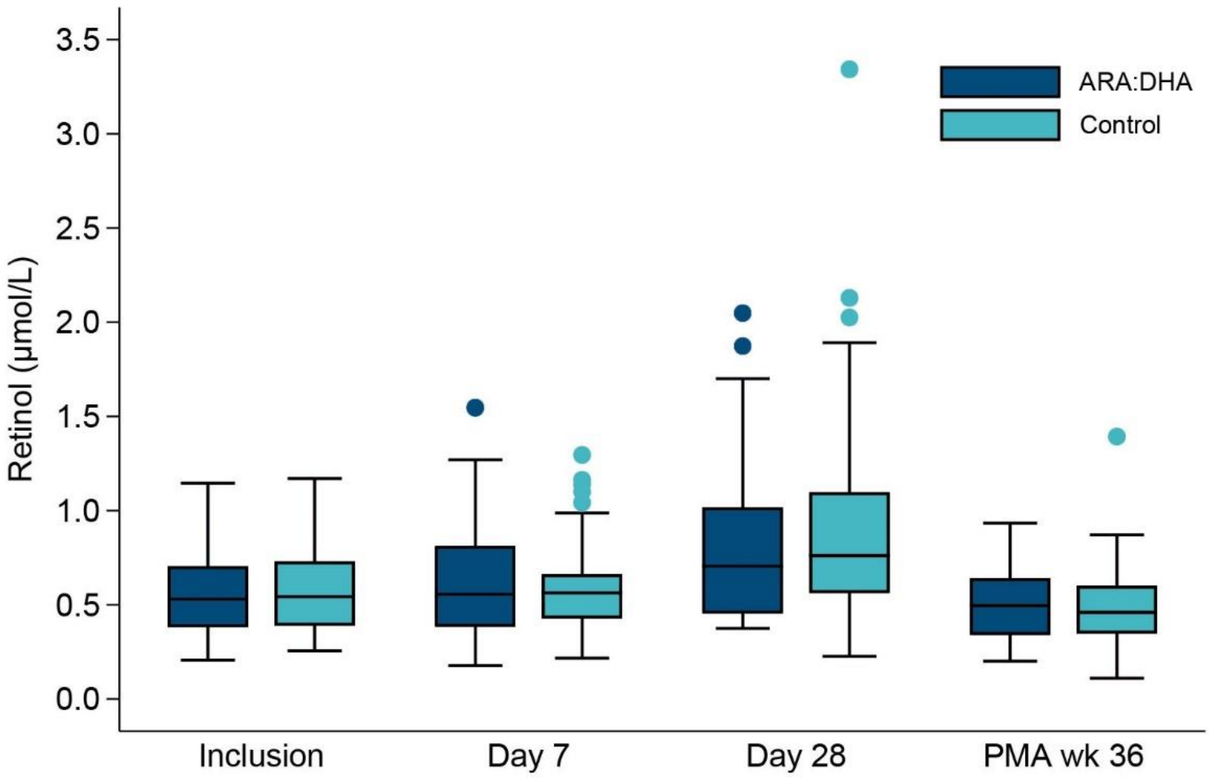

Figure S1. Longitudinal measures of retinol concentrations according to randomization group. Number of infants with retinol measurements at each study time point: Inclusion *n*=99, Day 7 *n*=96, Day 28 *n*=83 and 36 weeks PMA *n*=86. PMA, postmenstrual age.

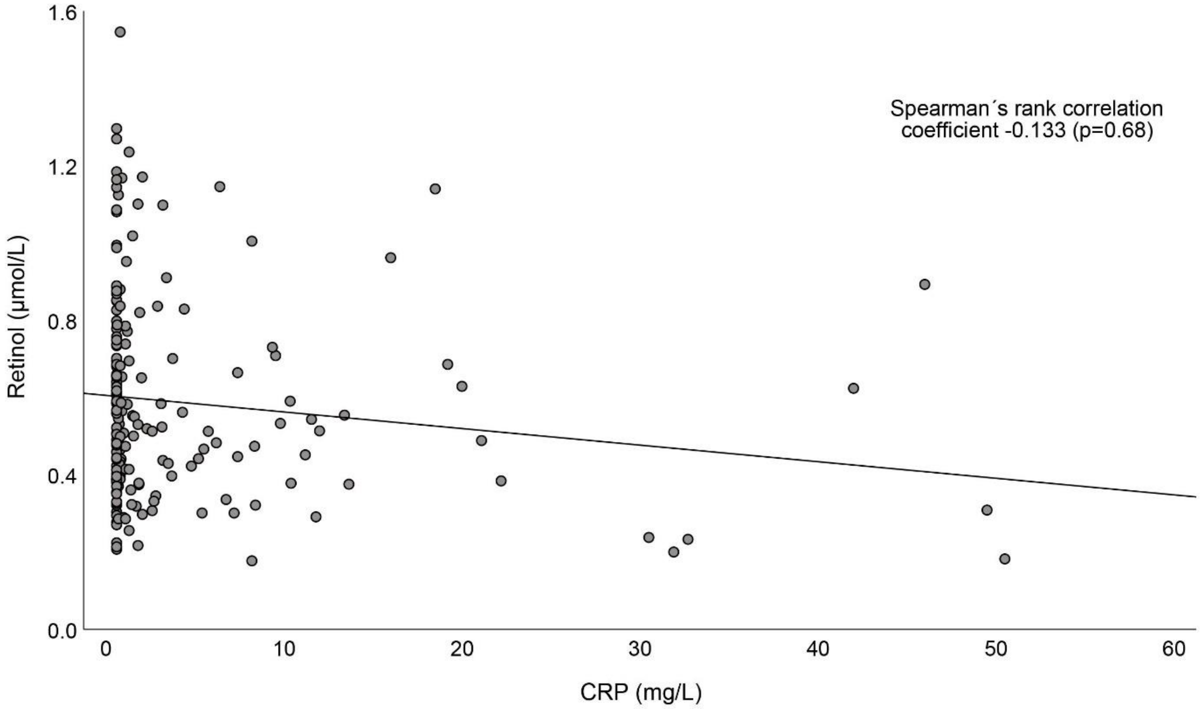

Figure S2. Associations between first week CRP levels and retinol concentrations (*n*=188). CRP, C-reactive protein.

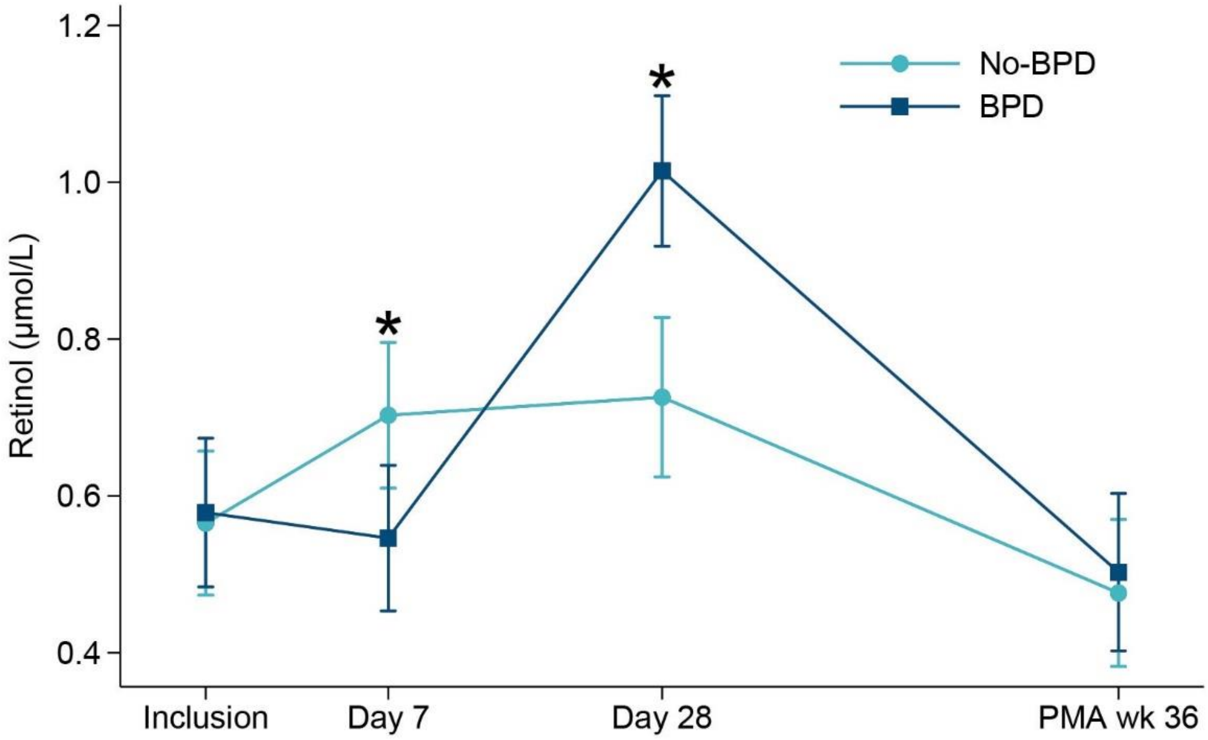

Figure S3. Longitudinal measures of retinol concentrations according to BPD group. Data are in means and error bars; 95% CI. Day 7 (p=0.019) and Day 28 (p<0.001). Number of infants with retinol measurements at each study time point: Inclusion *n*=99, Day 7 *n*=96, Day 28 *n*=83 and 36 weeks PMA *n*=86. BPD, bronchopulmonary dysplasia; PMA, postmenstrual age.
